# Risk Factors for Readmission Following Elderly Low Energy Pelvis Fractures

**DOI:** 10.1177/21514593251350498

**Published:** 2025-06-18

**Authors:** Sean Thomas, Avinaash Korrapati, Brendan O’Leary, Cooper Haaland, Alexandra K. Schwartz, William T. Kent

**Affiliations:** 1 University of California San Diego School of Medicine, La Jolla, CA, USA; 2 Department of Orthopaedic Surgery, 8784 University of California San Diego, CA, USA

**Keywords:** pelvic fracture, readmission, discharge disposition, fragility fractures, osteoporosis

## Abstract

**Introduction:**

Fragility fractures of the pelvis (FFP) are associated with loss of mobility and significant mortality in elderly patients. The purpose of this study was to assess the 60-day readmission rates following FFP and to identify what factors may predict readmission.

**Methods:**

Six hundred thirty-one patients over 65 years of age presenting for FFP to a level 1 trauma center between 2010 and 2022 were reviewed. The chi-square test of independence and logistic regression were performed to identify factors associated with readmission.

**Results:**

One hundred and thirty-six patients met inclusion criteria. Of these, 31 (23%) returned to the Emergency Department (ED) within 60 days of discharge and 16 (12%) were readmitted. Chronic obstructive pulmonary disease (COPD) (OR = 3.30, *P* = .01), discharge home instead of to a skilled nursing facility (SNF) (OR = 2.75, *P* = .01), discharge home from the ED instead of admission to the hospital (OR = 2.95, *P* = .009), and an American Society of Anesthesiologists (ASA) score ≥4 (OR = 5.14, *P* = .03) were all associated with return to the ED. Patients who were able to ambulate in the ED were less likely to be admitted to the hospital (OR = 0.05, *P* < .001) and more likely to return to the ED within 60 days (OR = 4.52, *P* = .03).

**Conclusion:**

Return to the ED and readmission following FFP is common, with an incidence of 23% and 12% in our cohort. Patients who were not admitted as an inpatient after their initial presentation, and patients who were discharged home instead of to a SNF, both had a higher risk of repeat presentation within 60 days of discharge. Additionally, ambulation by patients in the ED may provide false reassurance, as these patients were less likely to be admitted as an inpatient, but more likely to subsequently return within 60 days.

## Introduction

Fragility fractures of the pelvis (FFP) typically result from low energy trauma in patients with osteoporosis and are of rising incidence in an aging population. While age-related osteoporosis is the most common underlying etiology of FFP, vitamin-D deficiency, corticosteroid use, and previous long-term immobilization are also known risk factors.^1^ Patients with FFP often present with acute onset pain and difficulty with ambulation. Given the often minimally displaced stable fracture pattern and increased risk of surgical complications in elderly populations, most patients are treated nonoperatively with physical therapy and early mobilization.^1,2^

Even stable pattern injuries can leave patients prone to functional decline and at risk for other adverse events including cardiopulmonary and thromboembolic incidents.^3^ In addition, elderly patients with FFP often have multiple preexisting comorbidities which may complicate recovery.^4^ The 90-day mortality rate post-FFP is high, ranging from 4-24%, and many patients develop persistent ambulation deficits.^3,5^ Given these ambulation deficits, and the fact that elderly patients may lack adequate social support, FFPs are associated with a significant decline in quality of life.^4^

Optimizing treatment and support systems for patients with FFP is critical, as the healthcare burden associated with these injuries is expected to rise with an aging population. Between 1993 and 2010 there was a 24% increase in the incidence of FFP, and from 2012 to 2019 the rate of fragility pubic rami fractures increased 7-fold in males and 5-fold in females.^1,6^ This trend is expected to continue, with projections showing a rise in FFP through 2025.^7^

In the management of FFP, there exists what has been described as a “care conundrum”.^2^ Patients with FFP cannot be discharged home until they can ambulate safely, but they also may not require interventions warranting inpatient admission.^2^ Discharge disposition in this scenario relies on several factors, though no studies to date have examined the impact of this decision on readmission rates or repeat presentations to the Emergency Department (ED). In addition, no studies have determined what impact comorbidities may have on readmission rates in patients with FFP. The purpose of this study was to quantify the 60-day readmission rate in patients with FFP and to determine how preexisting comorbidities and discharge disposition impact readmission rates in these patients.

## Materials and Methods

After Institutional Review Board (IRB) approval, a retrospective review was performed on all patients with pelvis fractures, identified by Current Procedural Terminology (CPT) coding, from August 2010 through February 2022 at a regional level 1 trauma center. Inclusion criteria included all patients greater than 65 years of age presenting for FFP. Exclusion criteria included patients with less than 60 days of follow-up, patients with high-energy mechanisms of injury, patients with subacute or chronic pelvis fractures, and patients with concomitant lower extremity injuries precluding weight-bearing.

Medical records were reviewed to obtain demographic data, medical comorbidities, mechanism of injury, fracture characteristics, distance ambulated in the ED, discharge disposition, and readmission or repeat presentation to the ED within 60 days of discharge for any reason. Medical records from outside institutions were included in review when available. The chi-square test of independence was performed on all categorical variables to identify factors associated with return to the ED within 60 days of discharge. Logistic regression was used to identify independent predictors for return to the ED while controlling for differences in patient age, gender, and preexisting medical conditions. All analysis was performed at a level of significance of 0.05.

## Results

From August 2010 to February 2022, 631 patients presenting with pelvis fractures were identified, 136 of which met inclusion criteria. One hundred and fifty-six patients were excluded for having fractures secondary to high-energy mechanisms, 124 for age less than 65 years, 122 for presenting with subacute/chronic pelvis fractures, 64 for having other lower extremity injuries precluding weight-bearing, and 29 for insufficient medical records.

The mean age was 81 +/− 8 years, and 80% (n = 109) of patients were female. Twenty three percent (n = 31) of patients returned to the ED within 60 days of discharge, and 12% (n = 16) were readmitted (Table 1). Average time between discharge and return to the ED was 21 days, and 66% of return ED visits occurred within 30 days of initial presentation. Of the patients who returned to the ED, 32% (n = 10) presented for reasons related to their initial fall (including pain in pelvis, repeat fall, and hip pain related to initial fall) (Table 2). All indications for ED presentations within 60 days are listed in Table 2.

**Table 1.** Patient Characteristics

Characteristic	Mean (SD)
Age	81 (8)
BMI	23 (4.4)
ASA	2.7 (0.7)
Charlson comorbidity index	5.7 (2.8)

	n (%)
Male	27 (20%)
Female	109 (80%)
Returned to ED within 60 days	31 (23%)
Readmission within 60 days	16 (12%)

BMI, body mass index; ASA, American Society of Anesthesiologists score; ED, emergency department.

**Table 2.** Cause for Return to the ED by Organ System

Organ system	Total	Percent
**Pulmonary**	**4**	**13%**
COPD	1	
Pneumonia	1	
Aspiration pneumonia	1	
Shortness of breath	1	
**Gastrointestinal**	**5**	**16%**
Nausea/vomiting	2	
GI bleed	1	
Bowel obstruction	1	
Perforated gastric ulcer	1	
**Cardiovascular**	**2**	**7%**
Paroxysmal atrial fibrillation	1	
Chest pain	1	
**Musculoskeletal**	**14**	**45%**
Pelvic pain	8	
Fall	1	
Hip pain	1	
Lumbar back pain	1	
Spinal stenosis	1	
Paresthesia in upper extremity	1	
Ulnar fracture	1	
**Hematologic**	**1**	**3%**
Pulmonary embolism	1	
**Other**	**5**	**16%**
Displacement of PICC	1	
PTSD	1	
Hyperkalemia	1	
Cellulitis	1	
Altered mental status	1	
**Total**	**31**	

COPD, chronic obstructive pulmonary disease; GI, gastrointestinal; PICC, peripherally inserted central catheter; PTSD, post-traumatic stress disorder; ED, emergency department.

Factors associated with return to the ED within 60 days of discharge included history of COPD (OR = 3.30, *P* = .01), discharge home instead of to a SNF (OR = 2.75, *P* = .01), discharge home from the ED instead of admission to the hospital (OR = 2.95, *P* = .009), and ASA ≥4 (OR = 5.14, *P* = .03) (Table 3). Patients who ambulated in the ED were less likely to be admitted to the hospital (OR = 0.05, *P* < .001) and more likely to return to the ED within 60 days (OR = 4.52, *P* = .03). A complete list of odds ratios related to ED readmissions are listed in Table 3. Of these factors, discharge home from the ED over admission to the hospital (OR = 1.23, *P* = .008) and discharge home instead of to a SNF (OR = 1.22, *P* = .03) both were found to be independent predictors for return to the ED within 60 days of discharge.

**Table 3.** Factors Associated With Return to the ED Within 60 Days of Discharge

Factor	Odds ratio	95% confidence		*P* value
Sent home from ED vs admit from ED	2.95	1.28	6.8	.009
Discharge home vs discharge to a SNF	2.75	1.15	6.5	.01
Ambulate in ED	4.52	1.83	11.2	.03
COPD	3.30	1.20	9.3	.01
ASA ≥4	5.14	1.03	25.6	.03
Hypertension	0.90	0.38	2.1	.8
Osteoporosis	0.71	0.31	1.6	.4
Steroids	3.88	0.90	16.5	.05
Diabetes	0.83	0.30	2.2	.7
Chronic kidney disease	1.45	0.54	3.9	.5
Congestive heart failure	1.73	0.66	4.5	.2
Age >80	1.30	0.56	3.0	.5

ED, emergency department; SNF, skilled nursing facility; COPD, chronic obstructive pulmonary disease; ASA, American Society of Anesthesiologists score.

## Discussion

Given the rising incidence of FFP and the high morbidity and mortality burden associated with these injuries, it is important to identify patients at risk for further complications following discharge. In our cohort, a considerable proportion of patients with FFP (23%) returned to the ED within 60 days, and 12% were readmitted. These findings are consistent with a previous report by Reito et al, which documented a 60-day readmission rate of 14.6% in patients with FFP.^3^ Notably, 16% of the patients in the Reito et al study who were readmitted within 60 days presented for repeat falls, compared to 13% in our cohort.

In addition to quantifying readmission rates, we present the first study to date identifying factors associated with repeat ED presentations in patients with FFP. Of note, discharge disposition was found to be a significant predictor of repeat presentation to the ED in our cohort. Patients with FFP who were discharged home were more likely to return to the ED within 60 days when compared to patients who were discharged to a SNF. Though not specific to FFP patients, Werner et al also demonstrated significantly higher 30-day readmission rates in geriatric patients who were discharged home vs to a SNF.^8^ Geriatric patients often have multiple comorbidities, as evidenced by the wide variation in cause for readmission in our cohort (Table 3). Additional support provided by SNFs in the form of continuous monitoring and management of chronic conditions may help prevent return to the ED in patients with FFP.

While we present data supporting the benefits of SNF placement in patients with FFP, it is worth noting that the current consensus on SNF placement in orthopaedic patients is inconclusive. Malik et al demonstrated higher readmission rates in patients with operative pelvic and acetabular fractures with non-home discharge destinations.^9^ Similarly, Pollock evaluated 1486 elderly patients with operative hip fractures and also found higher readmission rates in patients discharged to a SNF.^10^ While we present positive outcomes in patients discharged to SNFs, these results may not be pertinent to patients with other orthopaedic injuries.

In addition to SNF placement, admission status following initial presentation to the ED was also found to significantly impact readmission rates in FFP. Patients who were discharged from the ED were more likely to return within 60 days when compared to patients who were admitted as inpatient from the ED. Of note, half of the readmissions in this cohort were for chief complaints related to the initial pelvis fracture. Other medical causes for readmission in this cohort may also have been secondary to FFP, as issues with mobility and rehabilitation following fragility fractures have been shown to exacerbate preexisting medical conditions.^11^ These patients may have benefited from further rehabilitation and medical stabilization in the form of an inpatient admission, and these findings further strengthen the case for a conservative approach to discharge in patients with FFP.

Admission criteria in patients with FFP relies on several factors. With the priority in treatment being early mobilization, medically stable patients who demonstrate ambulation in the ED may not be viewed as a strong candidate for inpatient admission. In line with this reasoning, patients in our cohort who ambulated in the ED were less likely to be admitted as an inpatient. These patients, however, were also more likely to subsequently return to the ED within 60 days of discharge. In this scenario, early ambulation by patients in the ED may provide false reassurance, and overreliance on this metric may lead to the discharge of patients with active medical problems who would otherwise benefit from inpatient treatment.

In addition to discharge disposition, comorbidities were also found to play a role in readmission rates following FFP. In our cohort, patients with COPD or an ASA ≥4 were more likely to return to the ED within 60 days of discharge. Previous studies have demonstrated high readmission rates in hip fracture patients with preexisting pulmonary disease and high ASA scores. ASA scores have also been identified as a predictor for readmission in orthopaedic trauma patients.^11^ Increased readmission rates in these patients may be related to exacerbation of chronic conditions following FFP. As shown in our cohort, knowledge of these preexisting comorbidities may be a useful tool in identifying patients with increased risk of readmission.

This study is not without limitations. The retrospective nature of this research limits data collection to that which was previously recorded in patient charts and potential selection bias.

While all available medical records were reviewed to ensure accurate data collection, including medical records from outside institutions, it is possible that some readmissions to outside facilities were not identified in our analysis due to insufficient records. In addition, ambulation in the ED was used as a metric to predict for readmission in our cohort. Collection of this data relied on accurate documentation in the ED, and it is possible that variations in documentation influenced this metric. Lastly, a power analysis for sample size was not performed for this study.

## Conclusion

In conclusion, readmission to the ED within 60 days of discharge in patients with FFP is common, with an incidence of 23% in our cohort. Patients who were not admitted as an inpatient after their initial presentation, and patients who were discharged home instead of to a SNF, both had a higher risk of returning to the ED. Comorbidities, including history of COPD and an ASA score ≥4, were also found to be associated with readmission within 60 days. Finally, ambulation by patients in the ED may provide false reassurance, as these patients were less likely to be admitted as an inpatient and more likely to subsequently return to the ED within 60 days of discharge. Patients with FFP often have multiple comorbidities complicating recovery. Care should be taken when evaluating these patients prior to discharge, as discharge disposition and comorbidities may have a significant impact on readmission rates in these patients.

## Ethical Statement

This study received ethical approval from the UCSD IRB (approval # 811230), with the need for written informed consent waived.

## Data Availability

The datasets generated during and/or analyzed during the current study are available from the corresponding author on reasonable request.*
